# Gender differences in the association between smoking and the risk of suicide in depressed patients: a longitudinal national cohort study

**DOI:** 10.3389/fpsyt.2025.1564915

**Published:** 2025-10-21

**Authors:** Yoo Jin Jang, Hyewon Kim, Jin-Hyung Jung, Kyungdo Han, Hong Jin Jeon

**Affiliations:** ^1^ Department of Psychiatry, Samsung Medical Center, Sungkyunkwan University School of Medicine, Seoul, Republic of Korea; ^2^ Department of Psychiatry, Hallym University Sacred Heart Hospital, Anyang, Republic of Korea; ^3^ Samsung Biomedical Research Institute, Sungkyunkwan University School of Medicine, Suwon, Republic of Korea; ^4^ Department of Statistics and Actuarial Science, Soongsil University, Seoul, Republic of Korea; ^5^ Department of Health Sciences & Technology, Department of Medical Device Management & Research, and Department of Clinical Research Design & Evaluation, Samsung Advanced Institute for Health Sciences & Technology (SAIHST), Sungkyunkwan University, Seoul, Republic of Korea

**Keywords:** smoking, suicide, death by suicide, depression, gender difference

## Abstract

**Introduction:**

Although the gender differences in suicide and smoking are well-known, studies exploring the impact of gender on the relationship between smoking and suicide are limited. This population-based nationwide cohort study examines the association between smoking and suicide risk among1.8 million depressed patients, analyzed separately by gender.

**Methods:**

This longitudinal cohort study included 1,827,249 adults diagnosed with depression between 2010 and 2015 from the Korean National Health Insurance Service database. Smoking status (never, former, current) was self-reported during health screenings, and suicides were identified via national mortality records. Cox proportional hazards models adjusted for demographic, clinical, and psychiatric covariates assessed hazard ratios (HRs) for suicide risk. Subgroup analyses explored effect modifications by covariates, including age, income, and alcohol use.

**Results:**

Over a median follow-up of 6.8 years, 6,318 individuals (0.35%) died by suicide. Smoking was associated with increased suicide risk in both men and women, with a stronger association in women. Current smoking showed a higher risk in women (HR = 2.646, 95% CI: 2.287-3.062) compared to men (HR = 1.376, 95% CI: 1.277-1.483). In men, factors such as younger age and alcohol consumption intensified this association, whereas in women, low income was a significant modifier; the highest suicide risk was observed in low-income former smokers.

**Conclusion:**

Smoking is associated with increased suicide risk among individuals with depression, with notable gender differences in risk magnitude and modifying factors. These findings highlight the need for gender-specific suicide prevention strategies. Limitations include reliance on self-reported smoking data and lack of time-varying measures of exposure.

## 1 Introduction

Suicide represents a critical public health concern in South Korea. Although there has been a modest reduction in recent years, South Korea’s suicide rate continues to be the highest among Organization for Economic Cooperation and Development countries, standing at 25.2 per 100,000 individuals (1). Based on the 2022 Korean suicide statistics, psychiatric issues, particularly depression, make up the majority (39.4%) of the motivations behind suicides (2). This information underscores the need for targeted suicide prevention strategies for individuals with mental disorders.

One emerging risk factor for suicide is smoking. Numerous studies have established a correlation between smoking and increased risks of suicidal ideation, attempts, and behaviors, with some further demonstrating a dose–response relationship, where heavier or more frequent smoking is associated with greater suicide risk (3–5). This link has been confirmed by recent meta-analyses (6). Smoking is more common among patients with depression (7) and correlates with an increased risk of future suicide in this demographic (8).

Male and female smokers show distinct psychological and neurobiological profiles. Men typically smoke for nicotine reinforcement, while women are influenced by non-nicotine factors, with distinct neurochemical mechanisms in the nicotinic acetylcholine and dopamine receptor systems playing a role in smoking addiction and cessation success (9). These differences extend to smoking prevalence, with a substantial sex disparity. In South Korea, the smoking rates are significantly higher among men (39.7% in 2016) than among women (3.3% in 2016) (10). Despite these well-documented differences in smoking behavior, few studies have explored whether the association between smoking and suicide risk differs by gender. Most existing research has treated gender as a covariate or control variable, rather than exploring its role as an effect modifier.

To address this gap, we hypothesize that gender significantly modifies the relationship between smoking and suicide, and that these patterns will differ by key covariates such as age, income, and alcohol use. Therefore, this population-based nationwide cohort study aims to 1) investigate whether the association between smoking and suicide risk differs by sex among patients with depression, and 2) examine whether sociodemographic and clinical factors modify this association differently in men and women.

## 2 Materials and methods

### 2.1 Study population

As with our previous research, which explored the link between physical activity and cardiovascular risk in patients with depression (11), this study also focuses on the population of patients with depression registered in the National Health Insurance Service (NHIS) database of South Korea. The NHIS administers a universal health insurance program that covers 97% of South Koreans, with the remainder enrolled in the medical aid program. The NHIS database houses anonymized medical service claims data and supports the largest national health screening program in the world. In South Korea, whenever individuals receive medical services, including outpatient visits, hospitalizations, or prescriptions, healthcare providers are required to electronically submit diagnostic and treatment information to the NHIS for reimbursement. This system allows for passive and complete follow-up of clinical outcomes through automatically collected administrative data, without the need for direct contact with participants. Mortality data are additionally linked from Statistics Korea (KOSTAT).

**Figure 1:**
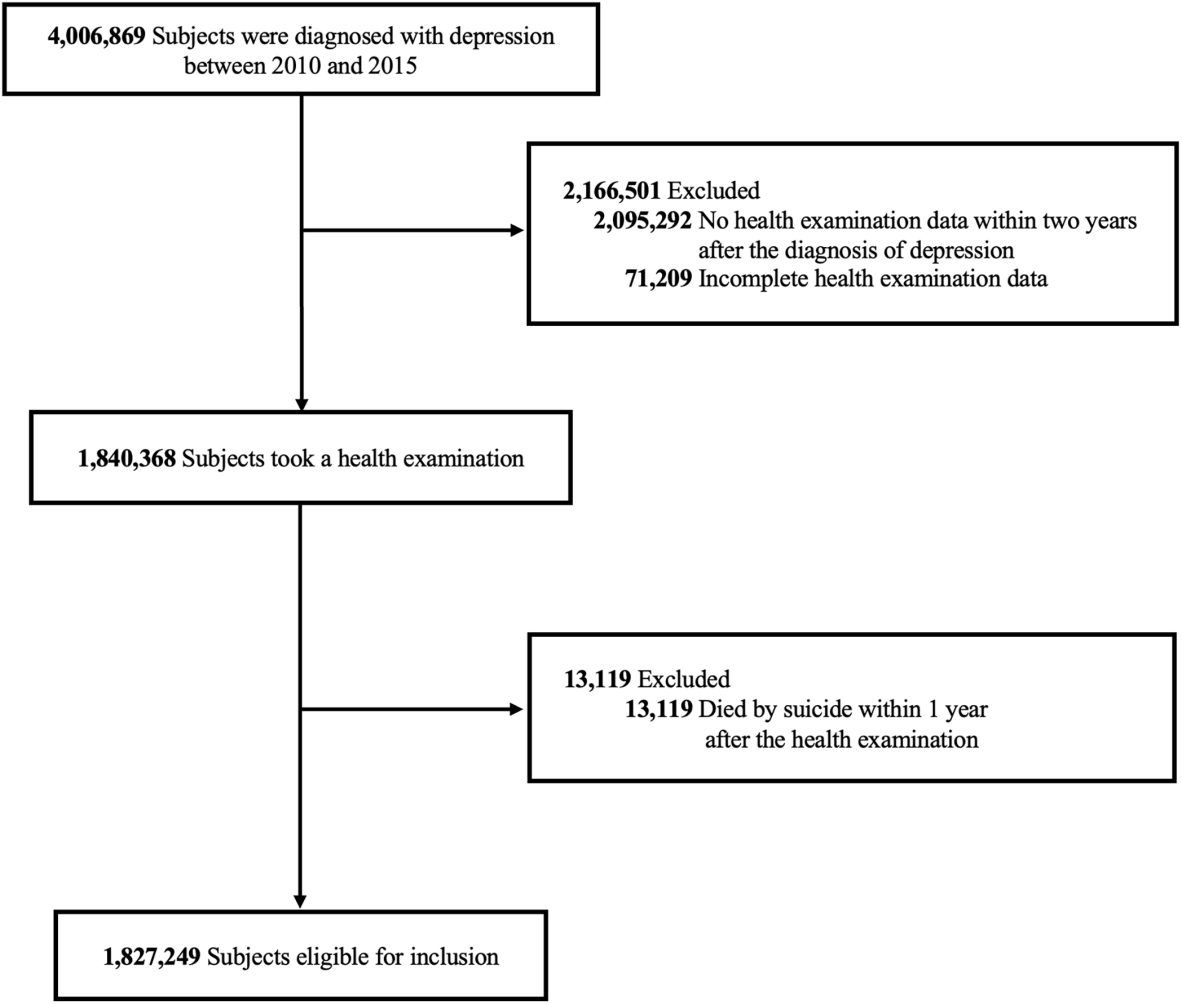
Selection of eligible study subjects.

**Figure 1** illustrated the flow diagram of the cohort selection process, including inclusion and exclusion criteria applied. A total of 4,006,869 individuals diagnosed with depression between January 1, 2010, and December 31, 2015, were identified using the International Classification of Disease, 10th Revision (ICD-10) codes F32 and F33. All diagnoses were made by licensed physicians, typically board-certified psychiatrists in clinical settings. Of these, 1,911,577 subjects who had undergone general health screenings within two years of their depression diagnosis were included. In South Korea, health screenings are typically conducted for individuals aged 20 and above; hence, the study only included those aged 20 and older. Due to incomplete health examination data, 71,209 subjects were excluded, resulting in 1,840,368 subjects with complete health examination data. A further 13,119 individuals who died by suicide within one year of the health examination were excluded to minimize reverse causality, enhancing the assessment of the long-term impact of smoking on suicide risk. These exclusions yielded a final sample of 1,827,249 subjects for analysis.

We followed the participants from the index date of January 1, 2010, until December 31, 2022, or until a suicide occurred during this period, which defined the follow-up period. Patient death and the end of the follow-up period were considered censoring events. Due to its retrospective design and the utilization of de-identified data, this study received an exemption from review by the Institutional Review Board of Samsung Medical Center (No. SMC 2022-10-140).

### 2.2 Smoking status

Participants’ smoking status was assessed through a self-administered questionnaire during health screenings conducted subsequent to their depression diagnosis. We classified participants into three groups based on their smoking status: never smoker, former smoker, or current smoker. The never smoker group was defined as those who indicated they had never smoked in their lifetime during the health screening. The former smoker group included individuals who had previously smoked but had quit, while the current smoker group consisted of those who reported they were actively smoking. These categories were selected to differentiate various levels of smoking exposure and evaluate their potential effects on health outcomes. Additionally, current and former smokers provided details about the total duration of smoking (in years) and the quantity smoked (in packs) to more precisely quantify smoking exposure and assess its potential impact on health outcomes.

### 2.3 Death by suicide

The primary outcome measured was the incidence of death by suicide during the follow-up period. Suicides were identified using the Korean cause of death data, which were linked to KOSTAT database. In this database, deaths by suicide were classified under the codes X60-X84.

### 2.4 Covariates

Low income was defined as below 25% of the national average, calculated from health insurance payments based on income levels in South Korea and encompassing those receiving medical aid. Alcohol consumption data were gathered through a self-administered questionnaire during post-diagnosis health screenings, classifying participants into ‘yes’ or ‘no’ categories based on whether their daily intake exceeded 0 grams of pure alcohol. Regular physical activity was engaging in moderate exercise on ≥ 5 days per week or vigorous exercise on ≥ 3 days per week. Body mass index (BMI) was derived from weight and height measurements, with obesity categorized as a BMI ≥ 25 kg/m². Data on glomerular filtration rate, systolic and diastolic blood pressure, and total cholesterol levels were also collected. Conditions such as diabetes mellitus (ICD-10 codes E11-E14 with related medication claims or fasting glucose ≥ 126 mg/dL), hypertension (ICD-10 codes I10-I15 with use of antihypertensive medications or blood pressure ≥140/90 mmHg), dyslipidemia (ICD-10 code E78 with antidyslipidemic medication claims or total cholesterol ≥ 240 mg/dL), and chronic kidney disease (estimated glomerular filtration rate < 60 mL/min/1.73m2) were recorded.

Data on comorbid psychiatric conditions and concomitant psychotropic medication utilization within one year following the onset of depression were obtained from insurance claims. Comorbid psychiatric conditions included schizophrenia spectrum disorders (ICD-10 codes F20-29), bipolar and related disorders (primary diagnoses for ICD-10 codes F30, F31, F34.0), anxiety disorders (primary diagnoses for ICD-10 codes F40, F41), obsessive-compulsive disorder (ICD-10 code F42), substance use disorder (ICD-10 codes F10-F19, including alcohol), intellectual developmental disorder (ICD-10 codes F70–79 or main disability cause ‘Intellectual Disability (06)’), major neurocognitive disorder (ICD-10 codes F00–03, G30–31), insomnia disorder (ICD-10 codes G47.0 and F51.0), alcohol use disorder (ICD-10 code F10), eating disorders (ICD-10 code F50), personality disorders (ICD-10 codes F60 and F61), autism spectrum disorder (ICD-10 code F84 or main disability cause ‘Autistic Disorder (07)’), post-traumatic stress disorder (PTSD) (ICD-10 code F43.1), attention-deficit hyperactivity disorder (ADHD) (ICD-10 code F90), and traumatic brain injury (ICD-10 code S06). Concomitant psychotropic medications included antidepressants, antipsychotics, anxiolytics, mood stabilizers, psychostimulants, and Z-drugs, as detailed in **Supplementary Table 1**.

### 2.5 Statistical analysis

Baseline characteristics of the study population were summarized by sex. Continuous variables were presented as means with standard deviations, while categorical variables were denoted as frequencies and percentages. One-way analysis of variance and chi-square tests were utilized for group comparisons. Cox proportional hazards regression models were estimated hazard ratios (HRs) and 95% confidence intervals (CIs) for the association between changes in smoking status and suicide risk. Incidence rates were expressed per 1,000 person-years.

Five models were developed for this analysis: Model 1 was non-adjusted; Model 2 was adjusted for age, low income, alcohol consumption, and regular physical activity to address basic demographic and lifestyle factors; Model 3 incorporated all variables from Model 2 plus comorbid physical illnesses such as obesity, diabetes mellitus, hypertension, dyslipidemia, and chronic kidney disease; Model 4 added to Model 3 comorbid psychiatric conditions including schizophrenia spectrum disorder, bipolar and related disorders, anxiety disorders, obsessive-compulsive disorder, substance use disorder, intellectual developmental disorder, major neurocognitive disorder, insomnia disorder, alcohol use disorder, eating disorder, personality disorder, autism spectrum disorder, PTSD, ADHD, and traumatic brain injury; and Model 5 integrated all variables from Model 4 along with concomitant psychotropic medications such as antidepressants, antipsychotics, anxiolytics, mood stabilizers, psychostimulants, and Z-drugs. The cumulative incidence of suicide during the follow-up period was estimated using Kaplan-Meier curves, stratified by smoking status, with differences assessed via the log-rank test.

Sensitivity analyses assessed the impact of cumulative smoking exposure post-depression diagnosis on suicide risk. Smoking exposure was quantified in pack-years (PYs), defined as the number of packs smoked per day times the years of smoking. Participants identified as current or former smokers at the health examination were classified into groups of < 5 PYs and ≥ 5 PYs, reflecting the average cumulative smoking amount of approximately 5 PYs observed among female smokers in our sample. Cox proportional hazards models, with adjustments akin to those used in the main analysis, estimated HRs for each category.

Subgroup analyses were conducted to explore the influences of covariates on the association between smoking and suicide, incorporating interaction terms in the Cox models to check for potential effect modification. All analyses were conducted using SAS version 9.4 (SAS Institute Inc., Cary, NC, USA).

## 3 Results

### 3.1 Baseline characteristics of study population

**Table 1** summarized the baseline demographic and clinical characteristics of the study population by sex. Of the total 1,827,249 individuals with depression, 60.3% were women. The mean age was 56.02 years, with a majority aged between 40 and 64 years.

**Table 1:** Baseline demographic and clinical characteristics of participants by sex.

Variable	Total	Sex			
		Men	Women	P	
N	1,827,249	725,792 (39.72)	1,101,457 (60.28)	<.0001	
Smoking Status				<.0001	
Never	1,273,972 (69.72)	240,194 (33.09)	1,033,778 (93.86)		
Former	263,666 (14.43)	240,426 (33.13)	23,240 (2.11)		
Current	289,611 (15.85)	245,172 (33.78)	44,439 (4.03)		
Smoking Status with Cumulative Amount				<.0001
Never	1,273,972 (69.72)	240,194 (33.09)	1,033,778 (93.86)		
Former < 5 Pack-Years	49,898 (2.73)	35,556 (4.90)	14,342 (1.30)		
Former ≥ 5 Pack-Years	213,768 (11.70)	204,870 (28.23)	8,898 (0.81)		
Current < 5 Pack-Years	42,364 (2.32)	23,919 (3.30)	18,445 (1.67)		
Current ≥ 5 Pack-Years	247,247 (13.53)	221,253 (30.48)	25,994 (2.36)		
Age	56.02 ± 13.97	55.41 ± 14.32	56.42 ± 13.72	<.0001	
20–39 years	215,342 (11.79)	108,187 (14.91)	107,155 (9.73)		
40–64 years	1,092,394 (59.78)	410,135 (56.51)	682,259 (61.94)		
≥65 years	519,513 (28.43)	207,470 (28.59)	312,043 (28.33)		
Low Income	402,627 (22.03)	133,766 (18.43)	268,861 (24.41)	<.0001	
Alcohol Consumption	613,714 (33.59)	397,522 (54.77)	216,192 (19.63)	<.0001	
Regular Physical Activity	351,694 (19.25)	161,704 (22.28)	189,990 (17.25)	<.0001	
Obesity	621,093 (33.99)	271,633 (37.43)	349,460 (31.73)	<.0001	
Diabetes Mellitus	267,271 (14.63)	129,022 (17.78)	138,249 (12.55)	<.0001	
Hypertension	692,020 (37.87)	297,235 (40.95)	394,785 (35.84)	<.0001	
Dyslipidemia	576,957 (31.58)	209,610 (28.88)	367,347 (33.35)	<.0001	
Chronic Kidney Disease	127,793 (6.99)	44,698 (6.16)	83,095 (7.54)	<.0001	
Comorbid Psychiatric Illnesses	661,721 (36.21)	259,790 (35.79)	401,931 (36.49)	<.0001	
Schizophrenia	36,855 (2.02)	17,689 (2.44)	19,166 (1.74)	<.0001	
Bipolar Disorder	12,561 (0.69)	5,518 (0.76)	7,043 (0.64)	<.0001	
Anxiety Disorder	178,102 (9.75)	67,531 (9.30)	110,571 (10.04)	<.0001	
Obsessive-Compulsive Disorder	10,652 (0.58)	5,285 (0.73)	5,367 (0.49)	<.0001	
Substance Use Disorder	47,034 (2.57)	36,153 (4.98)	10,881 (0.99)	<.0001	
Intellectual Developmental Disorder	5,893 (0.32)	3,259 (0.45)	2,634 (0.24)	<.0001	
Dementia	49,635 (2.72)	18,418 (2.54)	31,217 (2.83)	<.0001	
Insomnia disorder	453,671 (24.83)	168,056 (23.15)	285,615 (25.93)	<.0001	
Alcohol Use Disorder	43,258 (2.37)	34,278 (4.72)	8,980 (0.82)	<.0001	
Eating Disorder	5,620 (0.31)	1,348 (0.19)	4,272 (0.39)	<.0001	
Personality Disorder	4,029 (0.22)	2,189 (0.30)	1,840 (0.17)	<.0001	
Autism Spectrum Disorder	298 (0.02)	225 (0.03)	73 (0.01)	<.0001	
Post-Traumatic Stress Disorder	5,859 (0.32)	2,303 (0.32)	3,556 (0.32)	0.5171	
Attention-Deficit Hyperactivity Disorder	1,836 (0.10)	1,074 (0.15)	762 (0.07)	<.0001	
Traumatic Brain Injury	55,645 (3.05)	25,212 (3.47)	30,433 (2.76)	<.0001	
Psychotropic Medications	1,769,682 (96.85)	699,149 (96.33)	1,070,533 (97.19)	<.0001	
Antidepressants	1,486,224 (81.34)	589,824 (81.27)	896,400 (81.38)	0.0473	
Antipsychotics	138,998 (7.61)	61,959 (8.54)	77,039 (6.99)	<.0001	
Anxiolytics	1,332,338 (72.91)	491,806 (67.76)	840,532 (76.31)	<.0001	
Mood Stabilizers	98,009 (5.36)	44,677 (6.16)	53,332 (4.84)	<.0001	
Psychostimulants	53,393 (2.92)	15,860 (2.19)	37,533 (3.41)	<.0001	
Z-drugs	335,474 (18.36)	124,029 (17.09)	211,445 (19.20)	<.0001	
All-Cause Death	129,695 (7.10)	74,103 (10.21)	55,592 (5.05)	<.0001	
Death by Suicide	6,318 (0.35)	4,330 (0.60)	1,988 (0.18)	<.0001	
Follow-Up Duration					
Mean ± Standard Deviation	6.78 ± 1.99	6.61 ± 2.07	6.89 ± 1.93	<.0001	
Median (Quartile 1 - Quartile 3)	6.80 (5.25 - 8.30)	6.61 (5.14 - 8.17)	6.96 (5.37 - 8.41)	<.0001	

PY, pack-year.

Data are expressed as the mean standard deviation, or n (%).

*One-way analysis of variance or chi-square test was done. With p <0.05 indicating statistical significance.

Smoking status differed markedly by gender. Among men, current and former smokers each accounted for approximately one-third of the group, whereas nearly 94% of women were never smokers. When cumulative smoking amount was considered, over 90% of male current smokers had smoked ≥5 pack-years, compared to only 58.5% of female current smokers. Men had higher rates of alcohol consumption, regular physical activity, obesity, and metabolic comorbidities such as diabetes and hypertension. Women had a higher prevalence of most psychiatric conditions, including anxiety, insomnia, and eating disorders, and were more frequently prescribed psychotropic medications—particularly anxiolytics and Z-drugs.

### 3.2 Gender differences in the association of smoking status with suicide risk in depression

The association between smoking status and suicide risk was analyzed separately for men and women (**Table 2**). For men, never smokers served as the reference group with a suicide incidence rate of 0.850 per 1,000 person-years. Former smokers exhibited a consistently lower suicide risk than never smokers across all models, with the fully adjusted hazard ratio (Model 5) being 0.911 (95% CI: 0.842-0.986). Current smokers, on the other hand, had a significantly higher suicide risk than never smokers, with a fully adjusted hazard ratio of 1.376 (95% CI: 1.277-1.483). For women, former smokers displayed a significantly higher suicide risk than never smokers, with a fully adjusted hazard ratio (Model 5) of 2.111 (95% CI: 1.679-2.656). The highest suicide risk was observed among current smokers, with a fully adjusted hazard ratio of 2.646 (95% CI: 2.287-3.062).

**Table 2:** Cox regression analyses of the association between smoking status and suicide risk in depression, segregated by sex.

Outcome	Smoking in men			Smoking in women			P for interaction
	Never	Former	Current	Never	Former	Current	
N	240,194	240,426	245,172	1,033,778	23,240	44,439	
Suicide	1,336	1,188	1,806	1,701	77	210	
Duration	1,572,153.54	1,576,739.87	1,646,218.85	7,122,821.77	156,595.87	306,154.66	
Incidence Rate	0.850	0.753	1.097	0.239	0.492	0.686	
Model 1	1 (Reference)	0.886 (0.820, 0.958)	1.294 (1.205, 1.388)	1 (Reference)	2.055 (1.636, 2.582)	2.873 (2.489, 3.315)	<.0001
Model 2	1 (Reference)	0.921 (0.851, 0.996)	1.579 (1.467, 1.700)	1 (Reference)	2.472 (1.966, 3.109)	3.439 (2.975, 3.975)	<.0001
Model 3	1 (Reference)	0.927 (0.857, 1.003)	1.560 (1.449, 1.679)	1 (Reference)	2.439 (1.939, 3.067)	3.362 (2.908, 3.887)	<.0001
Model 4	1 (Reference)	0.910 (0.841, 0.985)	1.387 (1.287, 1.495)	1 (Reference)	2.177 (1.730, 2.738)	2.783 (2.405, 3.219)	<.0001
Model 5	1 (Reference)	0.911 (0.842, 0.986)	1.376 (1.277, 1.483)	1 (Reference)	2.111 (1.679, 2.656)	2.646 (2.287, 3.062)	<.0001

Incidence rates are per 1,000 person-years. Hazard ratios and 95% confidence intervals are provided for each model.

Model 1: Unadjusted.

Model 2: Adjusted for sex, age, low income, alcohol consumption, and regular physical activity.

Model 3: Includes adjustments from Model 2 plus obesity, diabetes mellitus, hypertension, dyslipidemia, and chronic kidney disease.

Model 4: Extends Model 3 by adjusting for schizophrenia, bipolar disorder, anxiety disorder, obsessive-compulsive disorder, substance use disorder, intellectual developmental disorder, dementia, insomnia disorder, alcohol use disorder, eating disorder, personality disorder, autism spectrum disorder, post-traumatic stress disorder, attention-deficit hyperactivity disorder, and traumatic brain injury.

Model 5: Adds to Model 4 adjustments for antidepressants, antipsychotics, anxiolytics, mood stabilizers, psychostimulants, and Z-drugs.

**Figure 2:**
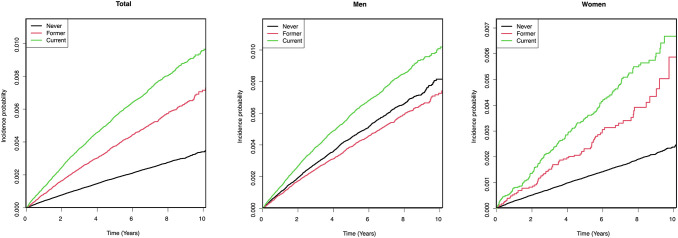
Adjusted Kaplan-Meier curves of cumulative incidence of suicide by smoking status in depressed patients.

**Figure 2** presents Kaplan–Meier survival curves showing the cumulative incidence of suicide according to smoking status. In both genders, current smokers exhibited the highest cumulative suicide risk, followed by former smokers and never smokers. The cumulative suicide risk increased more steeply by smoking status in women than in men, especially among current smokers. Among men, former smokers showed slightly lower cumulative risk than never smokers. The curves appeared nearly linear over time, indicating a steady accumulation of suicide risk throughout the follow-up period.

Sensitivity analyses examined the association between smoking status, cumulative smoking amount, and suicide risk for both sexes. Former and current smokers were categorized into those with less than 5 PYs and those with 5 or more PYs. The results demonstrate that the overall patterns observed in the initial analysis remain consistent, regardless of cumulative smoking amounts (**Supplementary Table 2**).

### 3.3 Sex differences in effect modification of covariates on the association between smoking status and suicide risk in depression

Subgroup analyses explored how various covariates affect the relationship between smoking status and suicide risk in men and women (**Table 3**). For men, younger age groups (20–39 years) who were current smokers had a significantly higher suicide risk (HR: 1.697, 95% CI: 1.329-2.169), while those aged 40–64 years (HR: 1.583, 95% CI: 1.421-1.762) also showed elevated risk with a significant interaction (p for interaction = 0.0002). Alcohol consumption increased the risk among current smokers, with non-drinkers (HR: 1.356, 95% CI: 1.224-1.502) and drinkers (HR: 1.563, 95% CI: 1.394-1.753) both exhibiting significant risks and a notable interaction (p for interaction = 0.0302). A borderline significant interaction was observed for obesity, with non-obese men having a higher risk (HR: 1.477, 95% CI: 1.350-1.615, p for interaction = 0.0538).

**Table 3:** Effect modification by demographic and clinical factors on the association between smoking status and suicide risk in depression.

Variable		Smoking status			
		Never	Former	Current	P for interaction
Men					
Age	20–39 years	1 (Reference)	0.975 (0.692, 1.374)	1.697 (1.329, 2.169)	**0.0002**
	40–64 years	1 (Reference)	0.868 (0.767, 0.982)	1.583 (1.421, 1.762)	
	65 years and older	1 (Reference)	0.981 (0.881, 1.091)	1.228 (1.083, 1.393)	
Income	Other	1 (Reference)	0.932 (0.853, 1.018)	1.439 (1.320, 1.570)	0.3371
	Lowest	1 (Reference)	0.859 (0.723, 1.021)	1.514 (1.310, 1.751)	
Alcohol Consumption	No	1 (Reference)	0.944 (0.853, 1.044)	1.356 (1.224, 1.502)	**0.0302**
	Yes	1 (Reference)	0.901 (0.793, 1.024)	1.563 (1.394, 1.753)	
Regular Physical Activity	No	1 (Reference)	0.908 (0.829, 0.994)	1.457 (1.339, 1.586)	0.9051
	Yes	1 (Reference)	0.944 (0.806, 1.106)	1.470 (1.252, 1.724)	
Obesity	No	1 (Reference)	0.974 (0.886, 1.070)	1.477 (1.350, 1.615)	0.0538
	Yes	1 (Reference)	0.800 (0.694, 0.922)	1.429 (1.252, 1.631)	
Diabetes Mellitus	No	1 (Reference)	0.932 (0.852, 1.020)	1.494 (1.372, 1.626)	0.4815
	Yes	1 (Reference)	0.866 (0.735, 1.020)	1.346 (1.153, 1.571)	
Comorbid Psychiatric Illness	No	1 (Reference)	0.911 (0.807, 1.028)	1.479 (1.319, 1.658)	0.9097
	Yes	1 (Reference)	0.920 (0.830, 1.020)	1.447 (1.311, 1.596)	
Psychotropic Medications	No	1 (Reference)	0.808 (0.654, 1.000)	1.407 (1.164, 1.701)	0.4447
	Yes	1 (Reference)	0.935 (0.859, 1.018)	1.470 (1.355, 1.595)	
Women					
Age	20–39 years	1 (Reference)	1.998 (1.265, 3.157)	2.833 (2.101, 3.819)	0.0804
	40–64 years	1 (Reference)	2.045 (1.534, 2.728)	2.210 (1.823, 2.678)	
	65 years and older	1 (Reference)	0.889 (0.398, 1.987)	1.557 (0.974, 2.489)	
Income	Other	1 (Reference)	1.551 (1.145, 2.102)	2.422 (2.019, 2.905)	**0.0436**
	Lowest	1 (Reference)	2.448 (1.713, 3.497)	1.933 (1.489, 2.509)	
Alcohol Consumption	No	1 (Reference)	1.899 (1.401, 2.576)	2.051 (1.659, 2.535)	0.4530
	Yes	1 (Reference)	1.809 (1.264, 2.588)	2.472 (1.978, 3.089)	
Regular Physical Activity	No	1 (Reference)	1.793 (1.385, 2.321)	2.133 (1.804, 2.521)	0.2636
	Yes	1 (Reference)	2.077 (1.233, 3.496)	2.896 (2.046, 4.098)	
Obesity	No	1 (Reference)	1.864 (1.433, 2.424)	2.381 (2.013, 2.816)	0.2644
	Yes	1 (Reference)	1.779 (1.095, 2.892)	1.732 (1.217, 2.463)	
Diabetes Mellitus	No	1 (Reference)	1.851 (1.448, 2.367)	2.362 (2.013, 2.772)	0.1401
	Yes	1 (Reference)	1.795 (0.886, 3.633)	1.384 (0.832, 2.304)	
Comorbid Psychiatric Illness	No	1 (Reference)	1.850 (1.207, 2.836)	2.859 (2.202, 3.713)	0.0924
	Yes	1 (Reference)	1.806 (1.372, 2.378)	2.017 (1.678, 2.424)	
Psychotropic Medications	No	1 (Reference)	1.509 (0.710, 3.207)	2.257 (1.439, 3.540)	0.8579
	Yes	1 (Reference)	1.884 (1.476, 2.403)	2.238 (1.904, 2.631)	

The model is adjusted for sex, age, low income, alcohol consumption, regular physical activity, as well as for medical condition including obesity, diabetes mellitus, hypertension, dyslipidemia, and chronic kidney disease. It also accounts for psychiatric conditions such as schizophrenia, bipolar disorder, anxiety disorder, obsessive-compulsive disorder, substance use disorder, intellectual developmental disorder, dementia, insomnia disorder, alcohol use disorder, eating disorder, personality disorder, autism spectrum disorder, post-traumatic stress disorder, attention-deficit hyperactivity disorder, and traumatic brain injury. Furthermore, the model includes adjustments for the use of psychotropic medications including antidepressants, antipsychotics, anxiolytics, mood stabilizers, psychostimulants, and Z-drugs.

The bold values indicate statistical significance at p < 0.05.

For women, low-income individuals with depression who were former smokers exhibited a higher suicide risk (HR: 2.448, 95% CI: 1.713-3.497) compared to current smokers (HR: 1.933, 95% CI: 1.489-2.509), while women from other income groups showed the highest suicide risk as current smokers (HR: 2.422, 95% CI: 2.019-2.905).

## 4 Discussion

This study revealed that smoking significantly affects suicide risk among individuals with depression, with former and current smokers exhibiting higher risks than never smokers. Smoking increased the risk of suicide for depressed patients of both genders, though women experienced a higher increase. In men, former smokers had a lower suicide risk than never smokers, whereas in women, the risk escalated from never smokers to former and current smokers. For men, factors such as younger age and alcohol consumption intensified the link between smoking and suicide. In women, low income altered the risk, with the highest risk observed among former smokers in the low-income group.

Our results underscore the gender differences in the relationship between smoking and suicide risk among depressed patients. Although men in our cohort had a higher overall smoking rate than women, the association between smoking and increased suicide risk was more pronounced in women. The differential effect by gender may arise from psychological variances between male and female smokers. Women tend to smoke to manage anxiety and depression and face more challenges in quitting due to changes in hypothalamic-pituitary-adrenocortical axis function during nicotine withdrawal (12). According to a study by Komiyama et al. on sex differences in nicotine dependency and depressive tendency among smokers, women smokers show higher depressive tendencies and have more difficulty in quitting smoking than men (13), potentially raising their suicide risk. In our sample, female smokers, whether current or former, had higher rates of comorbid psychiatric disorders than those who never smoked, whereas in men, rates of psychiatric comorbidities were similar across smoking statuses (**Supplementary Table 3**). This suggests that more severe psychiatric issues among female smokers may contribute to their increased suicide risk. Moreover, the stronger association observed in women may partly reflect cultural selection effects. In South Korea, where female smoking is socially stigmatized, women who smoke may represent a more psychologically vulnerable subgroup. In contrast, male smoking is more culturally normative and widespread, encompassing individuals with a broader range of psychological profiles (14). As a result, smoking among women may act as a marker of underlying vulnerability, contributing to the stronger link with suicide risk.

The pattern of suicide risk across smoking statuses differed notably by sex. Among men, former smokers exhibited a lower suicide risk than never smokers, whereas among women, suicide risk increased progressively from never to former to current smokers. The reduced suicide risk observed among male former smokers does not correspond with findings from earlier studies (3, 5, 15). Echeverria et al.’s meta-analysis suggested that former smokers have a higher relative risk of suicide than never smokers, although sex differences were not analyzed (15). Similarly, Miller et al.’s study, involving over 50,000 men, demonstrated a dose-dependent relationship between smoking and suicide, finding statistical significance only among heavy smokers (defined as individuals who smoke 15 or more cigarettes per day) (3). One plausible explanation is that male former smokers may be more proactive in managing their health (healthy quitter effect). In our study, male former smokers exhibited higher rates of physical comorbidities, such as obesity and diabetes, compared to never smokers and current smokers, a trend not observed in women (**Supplementary Table 3**). This suggests that men might quit smoking after being diagnosed with physical health issues to enhance their well-being. A recent nationwide cohort study identified a correlation between a healthy lifestyle and an increased sense of life purpose (16), which could act as a protective factor against suicide. Frequent hospital visits due to illness might also contribute to reducing the suicide risk (17). These visits enable regular monitoring of patients’ physical and mental health (18), facilitating the early detection of suicidal thoughts and behaviors. Additionally, they provide social interaction with healthcare staff and other patients, mitigating isolation and loneliness, and building trust and rapport with healthcare providers (19), which encourages patients to discuss their struggles and seek assistance. It is also possible that competing risks, such as increased mortality from physical illness in current smokers, may partially account for the relatively lower observed suicide risk in former smokers. Nonetheless, this interpretation should be made with caution given the observational nature of our study and the potential for residual confounding.

The factors that modified the association between smoking and suicide risk also differed by gender, suggesting distinct underlying mechanisms. Among men, younger age and alcohol consumption significantly amplified the suicide risk associated with smoking. These patterns suggest a stronger influence of neurobiological mechanisms, such as nicotine’s inhibition of monoamine oxidase and its impact on impulsivity (20), particularly in younger brains with greater plasticity (21). The pattern may also reflect a cohort effect. Younger male smokers in South Korea, where anti-smoking norms have strengthened since the mid-1990s, may represent a subgroup with higher psychological vulnerability (14, 22). The concurrent use of alcohol likely compounds these effects. Prior studies show a synergistic risk of suicide from smoking and alcohol use, particularly in men (23–25). In contrast, among women, low socioeconomic status significantly modified the smoking–suicide association. Former smokers in the lowest income group had a higher suicide risk than current smokers, possibly due to nicotine withdrawal exacerbated by the lack of protective resources. Withdrawal severity is often greater in women and linked to worse psychiatric symptoms (26, 27). Competing risk may also play a role. Current smokers in low-income groups may die earlier from other health conditions before suicide occurs (28). Overall, these findings suggest that while biological vulnerability may be more influential in men, psychosocial stressors may be more central in women. Although our study was not designed to directly investigate biological mechanisms, the observed gender-specific patterns underscore the need for future research, including neurobiological studies, to elucidate the underlying causal pathways.

Our findings highlight the need for gender-specific suicide prevention strategies among smokers with depression. Among men, younger individuals and those with alcohol use showed the greatest vulnerability. In our cohort, 14.9% of men were aged 20–39 and 54.8% reported alcohol consumption—subgroups in which smoking was most strongly associated with suicide (**Table 1**). Targeted interventions focusing on smoking cessation, impulse control, and alcohol use management in these men may yield substantial preventive benefits. Among women, low socioeconomic status emerged as a key modifier of the smoking–suicide association. Notably, former smokers in the lowest income group had a higher suicide risk than current smokers, suggesting unmet needs related to nicotine withdrawal and psychosocial stress. Enhancing cessation support and mental health care for low-income women could help mitigate this risk. Overall, tailoring tobacco control and suicide prevention efforts by gender and social context may enhance their effectiveness in high-risk psychiatric populations.

Our study has several limitations. First, smoking status was assessed through self-administered questionnaires at one health examination, which are subject to recall and social desirability biases (29, 30). This issue may be particularly salient among women, for whom smoking has been socially stigmatized in South Korea. The data captured average daily use and total years of smoking for conventional cigarettes only, without accounting for changes over time or the use of e-cigarettes and other tobacco products. These limitations may have affected the accuracy of exposure measurement. Although we calculated cumulative smoking exposure using pack-years and conducted a sensitivity analysis based on <5 PYs vs. ≥5 PYs, this measure may still fail to reflect the full complexity of individual smoking patterns, such as intermittent use or low-intensity daily smoking. Second, the use of baseline-only data limits the ability to capture dynamic changes in smoking behavior over time, such as cessation, relapse, or changes in intensity, which may differentially influence suicide risk (31, 32). Third, the exclusion of individuals who died by suicide within one year of the health examination aimed to minimize reverse causality but may introduce ‘over-exclusion’. Suicides often occur early in the diagnosis of depression (33), and these excluded individuals might differ from those who survived longer, potentially leading to a conservative estimation of suicide risk. Fourth, despite adjusting for numerous covariates, residual confounding due to unmeasured or inadequately measured variables may still affect the results. Factors such as genetic predisposition, depression severity, nicotine dependence, trauma history, and social support were not considered in the analysis. While we adjusted for multiple physical and psychiatric conditions, the interplay between these conditions and their cumulative impact on suicide risk relative to smoking status may not be fully captured.

## 5 Conclusion

This study offers an in-depth analysis of the association between smoking status and suicide risk, revealing significant gender differences in the relationships within a large cohort of individuals diagnosed with depression. Smoking elevates the risk of suicide for both genders, with a more pronounced risk in women. For men, the association between smoking and suicide risk is intensified by younger age and alcohol consumption. For women, low income is a significant factor, with the highest suicide risk observed in low-income former smokers. Our findings underscore the importance of targeted smoking cessation programs that incorporate robust mental health support, particularly for vulnerable populations. Future research should aim to elucidate sex-specific mechanisms underlying the smoking–suicide relationship.

## Data Availability

The data analyzed in this study is subject to the following licenses/restrictions: Korean National Health Insurance Service (NHIS). These data are not publicly accessible due to licensing restrictions for the current study. Requests to access these datasets should be directed to Kyungdo Han, hkd917@naver.com.
