# Supplementary material for: Gender differences in the association between smoking and the risk of suicide in depressed patients: a longitudinal national cohort study
Source: Front Psychiatry. 2025 Oct 21;16:1564915. doi: 10.3389/fpsyt.2025.1564915 (PMC12583080; doi:10.3389/fpsyt.2025.1564915)
Supplement: Supplementary file 1 [file Table1.docx]

Supplementary Material

# List of Concomitant Psychotropic Medications Used in the Study

|  | **Drug Code** | **Name** |
| --- | --- | --- |
| **Antidepressants** | |  |
|  | 107501ATB | amitriptyline hydrochloride 10mg |
|  | 107502ATB | amitriptyline hydrochloride 25mg |
|  | 107504ATB | amitriptyline hydrochloride 5mg |
|  | 108002ATB | amoxapine 50mg |
|  | 136301ACH | clomipramine hydrochloride 10mg |
|  | 136302ACH | clomipramine hydrochloride 25mg |
|  | 149203ATB | doxepin hydrochloride (as doxepin 3mg) |
|  | 149204ATB | doxepin hydrochloride (as doxepin 6mg) |
|  | 161501ACH | fluoxetine hydrochloride (as fluoxetine 10mg) |
|  | 161501ATB | fluoxetine hydrochloride (as fluoxetine 10mg) |
|  | 161502ACH | fluoxetine hydrochloride (as fluoxetine 20mg) |
|  | 161502ATB | fluoxetine hydrochloride (as fluoxetine 20mg) |
|  | 161502ATD | fluoxetine hydrochloride (as fluoxetine 20mg) |
|  | 162501ATB | fluvoxamine maleate 50mg |
|  | 162502ATB | fluvoxamine maleate 0.1g |
|  | 173701ATB | imipramine hydrochloride 25mg |
|  | 196201ATB | mirtazapine 15mg |
|  | 196201ATD | mirtazapine 15mg |
|  | 196202ATB | mirtazapine 30mg |
|  | 196202ATD | mirtazapine 30mg |
|  | 196203ATB | mirtazapine 45mg |
|  | 196204ATB | mirtazapine 7.5mg |
|  | 196204ATD | mirtazapine 7.5mg |
|  | 203401ATB | nortryptiline hydrochloride (as nortryptiline 10mg) |
|  | 203402ATB | nortryptiline hydrochloride (as nortryptiline 25mg) |
|  | 209301ATB | paroxetine hydrochloride (as paroxetine 10mg) |
|  | 209302ATB | paroxetine hydrochloride (as paroxetine 20mg) |
|  | 209304ATR | paroxetine hydrochloride (as paroxetine 12.5mg) |
|  | 209305ATR | paroxetine hydrochloride (as paroxetine 25mg) |
|  | 226401ATB | selegiline hydrochloride 5mg |
|  | 227001ATB | sertraline hydrochloride (as sertraline 50mg) |
|  | 227002ATB | sertraline hydrochloride (as sertraline 0.1g) |
|  | 227003ATB | sertraline hydrochloride (as sertraline 25mg) |
|  | 229601ATB | sodium tianeptine 12.5mg |
|  | 242901ACH | trazodone hydrochloride 25mg |
|  | 242901ATB | trazodone hydrochloride 25mg |
|  | 242902ATB | trazodone hydrochloride 50mg |
|  | 242903ATR | trazodone hydrochloride 75mg |
|  | 247502ACR | venlafaxine hydrochloride (as venlafaxine 75mg) |
|  | 247504ACR | venlafaxine hydrochloride (as venlafaxine 37.5mg) |
|  | 355801ACH | milnacipran hydrochloride 25mg |
|  | 355802ACH | milnacipran hydrochloride 50mg |
|  | 355803ACH | milnacipran hydrochloride 12.5mg |
|  | 428101ATB | bupropion hydrochloride 0.1g |
|  | 428102ATR | bupropion hydrochloride 0.15g |
|  | 428103ATR | bupropion hydrochloride 0.3g |
|  | 428301ATB | citalopram hydrobromide (as citalopram 20mg) |
|  | 474801ATB | escitalopram oxalate (as escitalopram 5mg) |
|  | 474802ATB | escitalopram oxalate (as escitalopram 10mg) |
|  | 474803ATB | escitalopram oxalate (as escitalopram 20mg) |
|  | 474804ATB | escitalopram oxalate (as escitalopram 15mg) |
|  | 495501ACE | duloxetine hydrochloride (as duloxetine 30mg) |
|  | 495501ATE | duloxetine hydrochloride (as duloxetine 30mg) |
|  | 495502ACE | duloxetine hydrochloride (as duloxetine 60mg) |
|  | 495502ATE | duloxetine hydrochloride (as duloxetine 60mg) |
|  | 613101ATB | agomelatine 25mg |
|  | 626401ATR | desvenlafaxine succinate monohydrate (as desvenlafaxine 50mg) |
|  | 626402ATR | desvenlafaxine succinate monohydrate (as desvenlafaxine 100mg) |
|  | 628501ATB | vortioxetine hydrobromide (as vortioxetine 5mg) |
|  | 628502ATB | vortioxetine hydrobromide (as vortioxetine 10mg) |
|  | 628504ATB | vortioxetine hydrobromide (as vortioxetine 20mg) |
|  | 687601ATR | desvenlafaxine benzoate (as desvenlafaxine 50mg) |
|  | 687602ATR | desvenlafaxine benzoate (as desvenlafaxine 0.1g) |
|  | 687701ATR | desvenlafaxine 50mg |
|  | 687702ATR | desvenlafaxine 0.1g |
|  | 687703ATR | desvenlafaxine 25mg |
| **Antipsychotics** | |  |
|  | 131901ATB | chlorpromazine hydrochloride 0.1g |
|  | 131905ATB | chlorpromazine hydrochloride 0.2g |
|  | 131908ATB | chlorpromazine hydrochloride 50mg |
|  | 137501ATB | clozapine 0.1g |
|  | 137502ATB | clozapine 25mg |
|  | 137503ATB | clozapine 50mg |
|  | 137504ATB | clozapine 0.2g |
|  | 167903ATB | haloperidol 1.5mg |
|  | 167904ATB | haloperidol 10mg |
|  | 167905ATB | haloperidol 20mg |
|  | 167906ATB | haloperidol 3mg |
|  | 167908ATB | haloperidol 5mg |
|  | 204001ATB | olanzapine 10mg |
|  | 204001ATD | olanzapine 10mg |
|  | 204002ATB | olanzapine 5mg |
|  | 204002ATD | olanzapine 5mg |
|  | 204004ATB | olanzapine 2.5mg |
|  | 204005ATB | olanzapine 15mg |
|  | 211401ATB | perphenazine 4mg |
|  | 212401ATB | pimozide 1mg |
|  | 212402ATB | pimozide 4mg |
|  | 224201ATB | risperidone 1mg |
|  | 224201ATD | risperidone 1mg |
|  | 224202ATB | risperidone 2mg |
|  | 224202ATD | risperidone 2mg |
|  | 224203ATB | risperidone 3mg |
|  | 224204ATB | risperidone 0.5mg |
|  | 233401ATB | sulpiride 0.2g |
|  | 250801ATB | zotepin 0.1g |
|  | 250802ATB | zotepin 25mg |
|  | 250803ATB | zotepin 50mg |
|  | 378601ATB | quetiapine fumarate (as quetiapine 25mg) |
|  | 378602ATB | quetiapine fumarate (as quetiapine 0.1g) |
|  | 378603ATB | quetiapine fumarate (as quetiapine 0.2g) |
|  | 378604ATB | quetiapine fumarate (as quetiapine 0.3g) |
|  | 378605ATB | quetiapine fumarate (as quetiapine 50mg) |
|  | 378605ATR | quetiapine fumarate (as quetiapine 50mg) |
|  | 378606ATR | quetiapine fumarate (as quetiapine 0.2g) |
|  | 378607ATR | quetiapine fumarate (as quetiapine 0.3g) |
|  | 378608ATR | quetiapine fumarate (as quetiapine 0.4g) |
|  | 378609ATR | quetiapine fumarate (as quetiapine 0.15g) |
|  | 378610ATB | quetiapine fumarate (as quetiapine 12.5mg) |
|  | 420002ATB | amisulpride 0.4g |
|  | 420003ATB | amisulpride 0.1g |
|  | 420004ATB | amisulpride 0.2g |
|  | 451501ATB | aripiprazole 10mg |
|  | 451501ATD | aripiprazole 10mg |
|  | 451502ATB | aripiprazole 15mg |
|  | 451502ATD | aripiprazole 15mg |
|  | 451503ATB | aripiprazole 5mg |
|  | 451504ATB | aripiprazole 2mg |
|  | 451505ATB | aripiprazole 30mg |
|  | 451508ATB | aripiprazole 1mg |
|  | 464901ACH | ziprasidone hydrochloride monohydrate (as ziprasidone 20mg) |
|  | 464902ACH | ziprasidone hydrochloride monohydrate (as ziprasidone 40mg) |
|  | 464903ACH | ziprasidone hydrochloride monohydrate (as ziprasidone 60mg) |
|  | 464904ACH | ziprasidone hydrochloride monohydrate (as ziprasidone 80mg) |
|  | 503201ATR | paliperidone 3mg |
|  | 503202ATR | paliperidone 6mg |
|  | 503203ATR | paliperidone 9mg |
|  | 511301ATB | blonanserin 2mg |
|  | 511302ATB | blonanserin 4mg |
|  | 511303ATB | blonanserin 8mg |
| **Anxiolytics** | |  |
|  | 105501ATB | alprazolam 1mg |
|  | 105502ATB | alprazolam 0.25mg |
|  | 105504ATB | alprazolam 0.4mg |
|  | 105505ATB | alprazolam 0.5mg |
|  | 105507ATB | alprazolam 0.125mg |
|  | 118501ATB | bromazepam 3mg |
|  | 120501ATB | buspirone hydrochloride 10mg |
|  | 120502ATB | buspirone hydrochloride 5mg |
|  | 120503ATB | buspirone hydrochloride 15mg |
|  | 131201ATB | chlordiazepoxide hydrochloride 10mg |
|  | 131202ATB | chlordiazepoxide hydrochloride 5mg |
|  | 135702ATB | clobazam 5mg |
|  | 136401ATB | clonazepam 0.5mg |
|  | 137302ATB | clotiazepam 5mg |
|  | 142901ATB | diazepam 10mg |
|  | 142902ATB | diazepam 2mg |
|  | 142903ATB | diazepam 5mg |
|  | 156201ATB | ethyl loflazepate 1mg |
|  | 156202ATB | ethyl loflazepate 2mg |
|  | 156501ATB | etizolam 1mg |
|  | 156502ATB | etizolam 0.5mg |
|  | 156503ATB | etizolam 0.25mg |
|  | 160601ATB | flunitrazepam 1mg |
|  | 161801ATB | flurazepam hydrochloride 15mg |
|  | 185501ATB | lorazepam 1mg |
|  | 185504ATB | lorazepam 0.5mg |
|  | 243501ATB | triazolam 0.125mg |
|  | 243502ATB | triazolam 0.25mg |
|  | 255800ATB | chlordiazepoxide 5mg |
| **Mood Stabilizers** | |  |
|  | 123130ASY | carbamazepine 2g(20mg/mL) |
|  | 123102ATB | carbamazepine 0.2g |
|  | 123102ATR | carbamazepine 0.2g |
|  | 123104ATR | carbamazepine 0.3g |
|  | 147701ATR | divalproex sodium (as divalproex 0.25g) |
|  | 147702ATR | divalproex sodium (as divalproex 0.5g) |
|  | 147801ACH | divalproex sodium particle (as divalproex 0.125g) |
|  | 181001ATB | lamotrigine 0.1g |
|  | 181002ATB | lamotrigine 25mg |
|  | 181003ATB | lamotrigine 50mg |
|  | 181004ATB | lamotrigine 5mg |
|  | 181005ATB | lamotrigine 2mg |
|  | 184701ATB | lithium carbonate 0.3g |
|  | 184702ATB | lithium carbonate 0.15g |
|  | 206330ASS | oxcarbazepine 6g(60mg/mL) |
|  | 206301ATB | oxcarbazepine 0.3g |
|  | 206302ATB | oxcarbazepine 0.6g |
|  | 206303ATB | oxcarbazepine 0.15g |
|  | 229734ASY | sodium valproate 9g(60mg/mL) |
|  | 229701ATR | sodium valproate 0.15g |
|  | 229703ATB | sodium valproate 0.2g |
|  | 229705ATR | sodium valproate 0.3g |
|  | 229706ATR | sodium valproate 0.5g |
|  | 229707ATR | sodium valproate 0.6g |
|  | 246901ATE | valproate magnesium 0.5g |
|  | 246902ATE | valproate magnesium 0.2g |
|  | 246903ATE | valproate magnesium 0.3g |
|  | 247001ACS | valproic acid 0.25g |
|  | 247002ACS | valproic acid 0.5g |
| **Psychostimulants** | |  |
|  | 193201ACR | methylphenidate hydrochloride 10mg |
|  | 193201ATB | methylphenidate hydrochloride 10mg |
|  | 193202ACR | methylphenidate hydrochloride 5mg |
|  | 193202ATB | methylphenidate hydrochloride 5mg |
|  | 193203ATR | methylphenidate hydrochloride 18mg |
|  | 193204ATR | methylphenidate hydrochloride 36mg |
|  | 193205ACR | methylphenidate hydrochloride 20mg |
|  | 193206ATR | methylphenidate hydrochloride 27mg |
|  | 193207ACR | methylphenidate hydrochloride 30mg |
|  | 193208ACR | methylphenidate hydrochloride 40mg |
|  | 193209ATR | methylphenidate hydrochloride 54mg |
|  | 193210ACR | methylphenidate hydrochloride 60mg |
|  | 193211ACR | methylphenidate hydrochloride 50mg |
|  | 251800ATB | caffeine anhydrous 0.1g |
|  | 439502ATB | modafinil 0.2g |
| **Z-drugs** |  |  |
|  | 250501ATB | zolpidem tartrate 10mg |
|  | 250502ATB | zolpidem tartrate 5mg |
|  | 250503ATR | zolpidem tartrate 6.25mg |
|  | 250504ATR | zolpidem tartrate 12.5mg |
|  | 680401ATB | eszopiclone 1mg |
|  | 680402ATB | eszopiclone 2mg |
|  | 680403ATB | eszopiclone 3mg |

# Association of Smoking Status with Cumulative Amount and Suicide Risk in Depressed Patients, Segregated by Sex

|  | **Smoking Status** | **N** | **Suicide** | **Duration** | **Incidence Rate** | **Model 1** | **Model 2** | **Model 3** | **Model 4** | **Model 5** |
| --- | --- | --- | --- | --- | --- | --- | --- | --- | --- | --- |
| Men | Never | 240,194 | 1,336 | 1,572,153.54 | 0.850 | 1 (Reference) | 1 (Reference) | 1 (Reference) | 1 (Reference) | 1 (Reference) |
|  | Former < 5 Pack-Years | 35,556 | 153 | 237,991.51 | 0.643 | 0.757 (0.641, 0.895) | 0.883 (0.747, 1.044) | 0.882 (0.745, 1.043) | 0.883 (0.746, 1.044) | 0.884 (0.747, 1.045) |
|  | Former ≥ 5 Pack-Years | 204,870 | 1,035 | 1,338,748.36 | 0.773 | 0.909 (0.838, 0.986) | 0.926 (0.854, 1.005) | 0.934 (0.861, 1.013) | 0.915 (0.843, 0.993) | 0.915 (0.843, 0.993) |
|  | Current < 5 Pack-Years | 23,919 | 125 | 162,479.02 | 0.769 | 0.908 (0.756, 1.090) | 1.302 (1.081, 1.569) | 1.290 (1.071, 1.553) | 1.193 (0.991, 1.437) | 1.175 (0.976, 1.415) |
|  | Current ≥ 5 Pack-Years | 221,253 | 1,681 | 1,483,739.83 | 1.133 | 1.336 (1.243, 1.435) | 1.602 (1.487, 1.726) | 1.583 (1.469, 1.705) | 1.404 (1.301, 1.514) | 1.393 (1.292, 1.503) |
| Women | Never | 1,033,778 | 1,701 | 7,122,821.77 | 0.239 | 1 (Reference) | 1 (Reference) | 1 (Reference) | 1 (Reference) | 1 (Reference) |
|  | Former < 5 Pack-Years | 14,342 | 51 | 97,476.75 | 0.523 | 2.188 (1.656, 2.891) | 2.874 (2.173, 3.801) | 2.804 (2.123, 3.708) | 2.531 (1.913, 3.348) | 2.451 (1.852, 3.242) |
|  | Former ≥ 5 Pack-Years | 8,898 | 26 | 59,119.12 | 0.440 | 1.836 (1.247, 2.705) | 1.936 (1.314, 2.852) | 1.937 (1.315, 2.854) | 1.705 (1.157, 2.513) | 1.657 (1.125, 2.442) |
|  | Current < 5 Pack-Years | 18,445 | 76 | 128,182.91 | 0.593 | 2.485 (1.975, 3.127) | 3.320 (2.633, 4.185) | 3.216 (2.551, 4.054) | 2.672 (2.119, 3.371) | 2.538 (2.012, 3.202) |
|  | Current ≥ 5 Pack-Years | 25,994 | 134 | 177,971.75 | 0.753 | 3.151 (2.643, 3.757) | 3.504 (2.936, 4.181) | 3.445 (2.887, 4.112) | 2.847 (2.385, 3.400) | 2.710 (2.269, 3.236) |
| p for interaction | |  |  |  |  | <.0001 | <.0001 | <.0001 | <.0001 | <.0001 |
| Incidence rates are per 1,000 person-years. Hazard ratios and 95% confidence intervals are provided for each model. Model 1: Unadjusted. Model 2: Adjusted for sex, age, low income, alcohol consumption, and regular physical activity. Model 3: Model 2 + adjusted for obesity, diabetes mellitus, hypertension, dyslipidemia, and chronic kidney disease. Model 4: Model 3 + adjusted for schizophrenia, bipolar disorder, anxiety disorder, obsessive-compulsive disorder, substance use disorder, intellectual developmental disorder, dementia, insomnia disorder, alcohol use disorder, eating disorder, personality disorder, autism spectrum disorder, post-traumatic stress disorder, attention-deficit hyperactivity disorder, and traumatic brain injury. Model 5: Model 4 + adjusted for use of antidepressants, antipsychotics, anxiolytics, mood stabilizers, psychostimulants, and Z-drugs. | | | | | | | | | | |

# Baseline Demographic and Clinical Characteristics of Participants by Smoking Status, Segregated by Sex

|  | **Smoking Status in Men** | | | | **Smoking Status in Women** | | | |
| --- | --- | --- | --- | --- | --- | --- | --- | --- |
|  | **Never** | **Former** | **Current** | **p** | **Never** | **Former** | **Current** | **p** |
| N | 240,194 | 240,426 | 245,172 |  | 1,033,778 | 23,240 | 44,439 |  |
| Cumulative Smoking | . | 19.74 ± 17.91 | 20.53 ± 15.28 | . | . | 5.78 ± 8.87 | 8.41 ± 9.19 | . |
| 5 or More Pack-Years | . | 204,870 (85.21) | 221,253 (90.24) | . | . | 8,898 (38.29) | 25,994 (58.49) | . |
| Age | 58.08 ± 15.23 | 58.07 ± 12.98 | 50.19 ± 13.18 | <.0001 | 56.91 ± 13.57 | 48.86 ± 14.34 | 48.91 ± 13.40 | <.0001 |
| 20-39 Years | 33,608 (13.99) | 20,640 (8.58) | 53,939 (22.00) |  | 92,710 (8.97) | 5,430 (23.36) | 9,015 (20.29) |  |
| 40-64 Years | 114,648 (47.73) | 140,685 (58.51) | 154,802 (63.14) |  | 637,104 (61.63) | 14,695 (63.23) | 30,460 (68.54) |  |
| 65 Years and Older | 91,938 (38.28) | 79,101 (32.90) | 36,431 (14.86) |  | 303,964 (29.40) | 3,115 (13.40) | 4,964 (11.17) |  |
| Low Income | 43,252 (18.01) | 39,659 (16.50) | 50,855 (20.74) | <.0001 | 246,512 (23.85) | 6,964 (29.97) | 15,385 (34.62) | <.0001 |
| Alcohol Consumption | 94,469 (39.33) | 136,004 (56.57) | 167,049 (68.14) | <.0001 | 181,749 (17.58) | 11,101 (47.77) | 23,342 (52.53) | <.0001 |
| Regular Physical Activity | 53,945 (22.46) | 63,783 (26.53) | 43,976 (17.94) | <.0001 | 179,352 (17.35) | 4,214 (18.13) | 6,424 (14.46) | <.0001 |
| Obesity | 88,938 (37.03) | 95,829 (39.86) | 86,866 (35.43) | <.0001 | 330,884 (32.01) | 6,643 (28.58) | 11,933 (26.85) | <.0001 |
| Diabetes Mellitus | 42,999 (17.90) | 45,964 (19.12) | 40,059 (16.34) | <.0001 | 130,776 (12.65) | 2,382 (10.25) | 5,091 (11.46) | <.0001 |
| Hypertension | 105,886 (44.08) | 109,802 (45.67) | 81,547 (33.26) | <.0001 | 377,424 (36.51) | 5,878 (25.29) | 11,483 (25.84) | <.0001 |
| Dyslipidemia | 66,671 (27.76) | 78,960 (32.84) | 63,979 (26.10) | <.0001 | 347,867 (33.65) | 6,584 (28.33) | 12,896 (29.02) | <.0001 |
| Chronic Kidney Disease | 18,213 (7.58) | 17,126 (7.12) | 9,359 (3.82) | <.0001 | 79,556 (7.70) | 1,331 (5.73) | 2,208 (4.97) | <.0001 |
| Comorbid Psychiatric Illnesses | 85,812 (35.73) | 84,554 (35.17) | 89,424 (36.47) | <.0001 | 372,062 (35.99) | 9,725 (41.85) | 20,144 (45.33) | <.0001 |
| Schizophrenia | 6,285 (2.62) | 4,415 (1.84) | 6,989 (2.85) | <.0001 | 17,445 (1.69) | 552 (2.38) | 1,169 (2.63) | <.0001 |
| Bipolar Disorder | 1,752 (0.73) | 1,411 (0.59) | 2,355 (0.96) | <.0001 | 6,117 (0.59) | 281 (1.21) | 645 (1.45) | <.0001 |
| Anxiety Disorder | 22,258 (9.27) | 24,161 (10.05) | 21,112 (8.61) | <.0001 | 103,264 (9.99) | 2,506 (10.78) | 4,801 (10.80) | <.0001 |
| Obsessive-Compulsive Disorder | 1,835 (0.76) | 1,659 (0.69) | 1,791 (0.73) | 0.0105 | 4,883 (0.47) | 176 (0.76) | 308 (0.69) | <.0001 |
| Substance Use Disorder (including alcohol) | 6,433 (2.68) | 8,094 (3.37) | 21,626 (8.82) | <.0001 | 7,448 (0.72) | 814 (3.50) | 2,619 (5.89) | <.0001 |
| Intellectual Developmental Disorder | 2,201 (0.92) | 310 (0.13) | 748 (0.31) | <.0001 | 2,531 (0.24) | 31 (0.13) | 72 (0.16) | <.0001 |
| Dementia | 9,236 (3.85) | 6,352 (2.64) | 2,830 (1.15) | <.0001 | 30,283 (2.93) | 449 (1.93) | 485 (1.09) | <.0001 |
| Insomnia Disorder | 55,277 (23.01) | 56,166 (23.36) | 56,613 (23.09) | 0.0111 | 263,698 (25.51) | 6,892 (29.66) | 15,025 (33.81) | <.0001 |
| Alcohol Use Disorder | 6,103 (2.54) | 7,534 (3.13) | 20,641 (8.42) | <.0001 | 5,955 (0.58) | 709 (3.05) | 2,316 (5.21) | <.0001 |
| Eating Disorder | 513 (0.21) | 397 (0.17) | 438 (0.18) | 0.0003 | 3,857 (0.37) | 138 (0.59) | 277 (0.62) | <.0001 |
| Personality Disorder | 680 (0.28) | 531 (0.22) | 978 (0.40) | <.0001 | 1,532 (0.15) | 120 (0.52) | 188 (0.42) | <.0001 |
| Autism Spectrum Disorder | 197 (0.08) | 13 (0.01) | 15 (0.01) | <.0001 | 72 (0.01) | 1 (0.00) | 0 (0.00) | 0.1909 |
| Post-Traumatic Stress Disorder | 709 (0.30) | 728 (0.30) | 866 (0.35) | 0.0005 | 3,178 (0.31) | 117 (0.50) | 261 (0.59) | <.0001 |
| Attention-Deficit Hyperactivity Disorder | 395 (0.16) | 260 (0.11) | 419 (0.17) | <.0001 | 658 (0.06) | 45 (0.19) | 59 (0.13) | <.0001 |
| Traumatic Brain Injury | 8,506 (3.54) | 7,504 (3.12) | 9,202 (3.75) | <.0001 | 28,067 (2.71) | 665 (2.86) | 1,701 (3.83) | <.0001 |
| Psychotropic Medications | 231,603 (96.42) | 231,830 (96.42) | 235,716 (96.14) | <.0001 | 1,004,620 (97.18) | 22,557 (97.06) | 43,356 (97.56) | <.0001 |
| Antidepressants | 193,622 (80.61) | 195,838 (81.45) | 200,364 (81.72) | <.0001 | 840,011 (81.26) | 19,222 (82.71) | 37,167 (83.64) | <.0001 |
| Antipsychotics | 20,862 (8.69) | 17,752 (7.38) | 23,345 (9.52) | <.0001 | 69,985 (6.77) | 2,186 (9.41) | 4,868 (10.95) | <.0001 |
| Anxiolytics | 165,726 (69.00) | 164,512 (68.43) | 161,568 (65.90) | <.0001 | 787,886 (76.21) | 17,771 (76.47) | 34,875 (78.48) | <.0001 |
| Mood Stabilizers | 15,346 (6.39) | 13,777 (5.73) | 15,554 (6.34) | <.0001 | 49,331 (4.77) | 1,373 (5.91) | 2,628 (5.91) | <.0001 |
| Psychostimulants | 5,489 (2.29) | 5,302 (2.21) | 5,069 (2.07) | <.0001 | 35,027 (3.39) | 886 (3.81) | 1,620 (3.65) | <.0001 |
| Z-drugs | 40,140 (16.71) | 40,929 (17.02) | 42,960 (17.52) | <.0001 | 193,775 (18.74) | 5,354 (23.04) | 12,316 (27.71) | <.0001 |
| All-Cause Mortality | 28,523 (11.87) | 24,129 (10.04) | 21,451 (8.75) | <.0001 | 52,277 (5.06) | 1,096 (4.72) | 2,219 (4.99) | 0.0553 |
| Death by Suicide | 1,336 (0.56) | 1,188 (0.49) | 1,806 (0.74) | <.0001 | 1,701 (0.16) | 77 (0.33) | 210 (0.47) | <.0001 |
| Follow-Up Duration |  |  |  |  |  |  |  |  |
| Mean ± Standard Deviation | 6.55 ± 2.11 | 6.56 ± 2.05 | 6.71 ± 2.03 | <.0001 | 6.89 ± 1.93 | 6.74 ± 1.92 | 6.89 ± 1.92 | <.0001 |
| Median (Quartile 1 - Quartile 3) | 6.56 (5.10-8.14) | 6.51 (5.12-8.13) | 6.75 (5.20-8.22) | <.0001 | 6.96 (5.38-8.42) | 6.73 (5.20-8.20) | 7.02 (5.35-8.40) | <.0001 |
| Abbreviation: Data are expressed as mean ± standard deviation, or n (%). *One-way analysis of variance or chi-square test was conducted, with p <0.05 indicating statistical significance. | | | | | | | | |
